# A way to reuse petrochemical wastes through the partial substitution of cement by spent cracking catalyst and its effect on cement pastes

**DOI:** 10.1038/s41598-025-33310-1

**Published:** 2025-12-30

**Authors:** Paweł Niewiadomski, Michał Cisiński, Barbara Batog, Agnieszka Chowaniec-Michalak, Elżbieta Janowska-Renkas, Martyna Nieświec

**Affiliations:** 1 Department of Materials Engineering and Construction Processes, Wroclaw University of Science and Technology, Wybrzeze Wyspianskiego 27, Wroclaw, 50-370 Poland; 2 Department of Building Materials Engineering, Opole University of Technology, Katowicka 48, Opole, 45-061 Poland; 3 Betotech Technology Center, Heidelberg Materials Polska, Rozdzienskiego 14, Dabrowa Gornicza, 41- 306 Poland; 4 Department of Environmental Engineering, Opole University of Technology, Mikolajczyka 5, Opole, 45-271 Poland

**Keywords:** Spent cracking catalyst, Oil refinery wastes, Cement paste, Supplementary cementitious materials, Pozzolanicity, Circular economy., Chemistry, Engineering, Environmental sciences, Materials science

## Abstract

This study examined the recycling of spent catalyst from Fluid Catalytic Cracking (FCC) process derived from Polish petroleum industry in cementitious materials. FCC is a basic process in converting crude oil into refinery products, utilizing zeolite catalyst. Nevertheless, owing to petroleum coke formation on its surface, such catalyst ends its life cycle in landfill as general solid waste (code 16 08 01) called spent Equilibrium Catalyst (E-Cat), which brings about serious environmental and health risks. Therefore, alternative approaches for E-Cat management are under development, one of which is recycling in cementitious materials. Nevertheless, there are still limitations in that scope arising from variable characteristics of E-Cat from different refineries and research gaps in terms of its influence on cementitious materials. Therefore, the current paper presents correlations between characteristics of Polish E-Cat, its superior pozzolanicity and impact of its usage as a 0–20% substitute of Portland cement, by mass, on fresh and hardened state performances of cement pastes at w/c ratios of 0.30, 0.35 and 0.40. It was demonstrated that the SiO_2_ and Al_2_O_3_ content in Polish E-Cat exceeds 87% of its mass, which contributes to its vital direct pozzolanic activity measured by Chapelle test (870.3 mg of Ca(OH)_2_/g E-Cat) and rapid, relevant and reliable (R^3^) test (497 J/g of E-Cat). Those performances contributed to the enhancement of pastes’ compressive strength for up to 16.7%, where the E-Cat was substituting 10–20% of cement, by mass, without application of any admixture. Simultaneously, other performances, such as consistency, flexural strength and water absorptivity were not significantly deteriorated. The partial substitution of cement with waste materials, such as E-Cat, brings about vital environmental benefits, which was additionally underlined in this paper.

## Introduction

### General overview on characteristics of E-Cat and its utilization as supplementary cementitious material

The reusing of oil refinery wastes in cementitious composites offers a significant chance for petrochemical and building sectors and for the environment in terms of resource efficiency, cost optimization, managing of open and historic landfills, reducing CO_2_ footprint and improving performances of concrete. The intense industrialization and production operations enforced by the recent economic growth have led to global environmental threads such as generation of waste materials, especially in developing countries^1^. As so to proceed further progress in the sustainable conditions, the 5R procedure given in the chronological order of refuse, reduce, reuse, reproduce and recycle is mostly recommended for managing waste materials^2^. Implementing it allows to initiate zero-waste and circular economy, shifting away from virgin resources by their substitution with waste materials to prolong their added value as far as possible. Such ideas are promoted and pushed forward with the progressively restrictive regulations such as Best Available Techniques Reference Documents (BREFs)^3^.

**Fig. 1:**
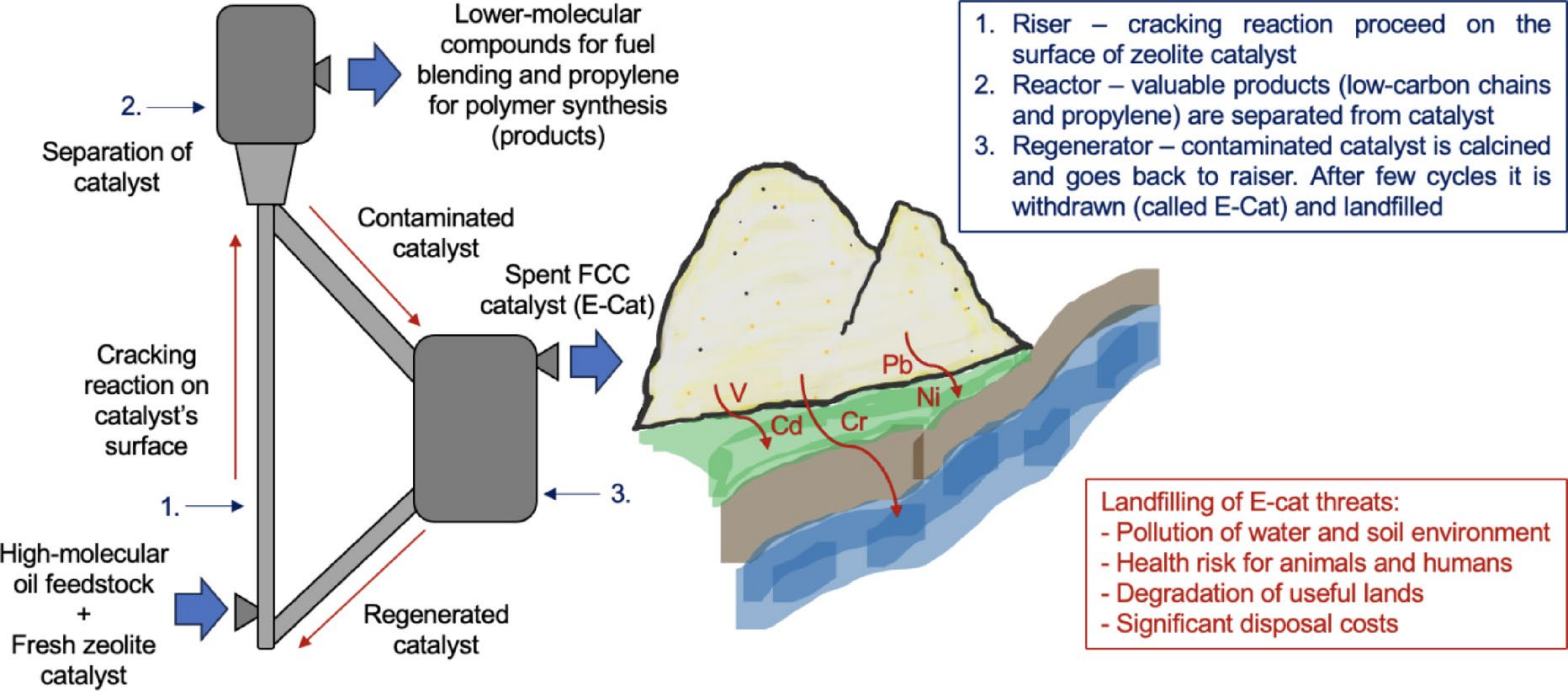
Principal of FCC process and most significant threats arising from landfilling of E-Cat.

Globally, significant amount of solid wastes are generated by petroleum industry^4^. Owing to the direct contact with crude oil resource during refining processes, it is particularly challenging to cope with spent catalysts among which approximately 70% is represented by spent catalyst from Fluid Catalytic Cracking (FCC) process^5^. The FCC is a key-important process in petroleum business, producing main feedstock for blending gasoline and source of olefines for polymer synthesis^6^. To achieve appropriate kinetics and efficiency parameters in industrial conditions, the cracking reaction, i.e. conversion of long hydrocarbon fractions to shorter chains, is promoted by the catalyst^6,7^ showing high chemical activity and selectivity. Commercially, FCC catalysts are a fine granules of synthesized Y-type zeolite known as faujasite spread on amorphous Al_2_O_3_ species^8^. After some time used in the process, such catalysts lose their activity due to mechanical degradation caused by attrition^9^ and, most notably, adsorption of petroleum coke deposits from crude oil on its surface^10^. These organic contaminants potentially comprise heavy metals, such as Cd, Cr, Pb^11^, Ni and V^12^. The regeneration of catalyst by roasting in high temperature in regenerator allows to maintain its performances and use it circularly in FCC process^13^. However, after some time becomes not sufficient or economically unjustified. Consequently, typically every three to four weeks^14^, such deactivated catalyst, called Equilibrium Catalyst (E-Cat)^6^, is withdrawn from FCC units and ends its life cycle in landfill as general solid waste (code 16 08 01)^5^. Such procedure, apart from generating high disposal costs (300 to 450 €/tone)^15^, provides serious environmental and health risk, as the chemical contaminants might undergo leaching by rainwater and pollute the environment^7^. The principal of FCC process together with the most significant threats arising from the landfilling of E-Cat are presented in Fig. 1.

The significance of such problem is enhanced while considering the scale of generation of E-Cat by worldwide petroleum business, which is estimated to vary from 200,000^16^, 400,000^17^, up to 840,000 tons annually^18^. According to papers emphasizing on FCC technology from these decade, the global conversion of oil feedstock in FCC units accounts for approximately 15,000,000 barrels per day^19^, while each converted barrel consumes 0.16 kg of catalyst^6^. Hence, by multiplying those values for 300 working days per year (typical assumption for continuous production process such as FCC having regard to cleaning stops, breakdowns and other issues), the factual global generation scale of E-Cat wastes confidently exceeds 700,000 tons per year. Moreover, as zeolite catalysts were used in more than 95% of worldwide FCC units already in the year 1962^20^, the global E-Cat deposition in landfills might be dozens of times higher, accounting in dozens of millions of tons. Finally, the generation of E-Cat is supposed to increase by approximately 5% in the next decade^14^ with the increasing market demand for petroleum products^21^. Such findings upscale the pressure for valorizing this material, not only regarding its future production, but most notably in terms of dozens of millions of tons disposed in currently open and historic landfills in the areas where petroleum industry operates or did it in the past. The stability of zeolites, especially those used by petrochemical industry for the FCC process, is considered to be very high in ambient environmental conditions^22^. Moreover, the organic hydrocarbons do not react with zeolites below the cracking temperatures, i.e. 500 °C^18^. Although there is no literature study comparing the reactivity of E-Cat wastes between different age of storage, the stability of zeolites might allow to assume that the properties of spent catalysts are not influenced over the storage time. However, another question appears in terms of variation and development of catalysts used in FCC process in terms of the recycling of E-Cat wastes in historic landfills. This matter is well described in the following contribution^23^.

Technically, E-Cat is an aluminosilicate material possessing substantial surface area of high-porous zeolite^21,24^. According to the literature, its specific surface area might be equal up to 118^25^ or even 250 m^2^/g^11^ measured by BET, depending, among others, on the particles’ fineness. Those properties are in line with the characteristics of artificial pozzolans^26,27^ and hence give grounds to consider utilization of E-Cat as a Supplementary Cementitious Material (SCMs) in cement and concrete products. However, at present, the E-Cat as a general waste material is denied from the use in cement (EN 197-1 standard^28^ and concrete (EN 206-1 standard^29^ from the regulation viewpoint. The potential for implementation of E-Cat might be achievable after the completion of all standardized measurements for cement and concrete incorporating E-Cat grains if the results match with the requirements of above mentioned standards based on the proposal for local technical assessments for this type of waste material. The valorization of E-Cat (e.g. through the calcination and grinding process) might allow to transform it into artificial pozzolan thereby eliminate organic carbons and hence the status of waste material.

Pozzolanic SCMs are inorganic powders which exhibit no binding properties itself but, when mixed with cement and water, undergo the so called pozzolanic reaction with Portlandite, a secondary product of hydration of clinker minerals, to form additional C-S-H-gel, enhancing concrete’s strength and durability^26,30^. In this regard, SCMs might be efficiently used as a partial substitute of Portland cement in cementitious materials, such as mortars and concretes. Such solution is highly recommended by various authorities, such as European Cement Association (CEMBUREAU), among the main measures to reduce carbon footprint in cement and concrete value chain^31^. This is because at the annual production scale of approximately 3.4 billion tons^32^, the manufacturing of Ordinary Portland Cement (OPC) directly causes 5–8% of global anthropogenic CO_2_ emission^33–35^. Currently, fly ash and blast furnace slag are the two most commonly used SCMs to produce blended cements with lower carbon footprint but their global availability is undoubtedly going to decline due to intensification of renewable energy and steel recycling^36^. Therefore, a lot of researchers around the world aim to propose new SCMs, especially locally available for cement industry. The open and historic landfills of aluminosilicate waste materials offer a high industrial and research potential for abovementioned application.

Moreover, besides the sustainable approach it offers, the idea of recycling E-Cat in cementitious materials is justified by some encouraging results from the literature. The experimental research carried out by Su et al.^11^, Lei et al.^14^, Payá et al.^37^, Al-Jabri et al.^38^, Tseng et al.^39^ and Vaičiukynienė et al.^40^ indicated that the substitution of up to 15–25% of cement in various cementitious mixes with E-Cat wastes from different oil refineries revealed positive impact on their compressive strength. Chen et al.^41^ demonstrated that replacement of 15% of OPC with E-Cat from Chinese petroleum corporation enhanced 28 days compressive strength of cement mortar for 36%.

These findings are attributable to pozzolanic properties of waste catalyst and hence to secondary C-S-H phase formed in cementitious system due to its reaction with Portlandite. The vital pozzolanic activity of E-Cat was confirmed directly by many researchers. For example, Nunes et al.^42^ evaluated that the amount of calcium hydroxide reacted with E-Cat (1540 mg of Ca(OH)_2_/g) was higher than typical values for different pozzolans, e.g. metakaolin (560–1140 mg of Ca(OH)_2_/g) and fly ashes (540–725 mg of Ca(OH)_2_/g).

Moreover, Su et al.^11^ and Al-Jabri et al.^38^ in their research proved that cementitious composites reveal the potential to immobilize heavy metals from E-Cat in their hardened-state structure through the leaching tests. This ensure that the risk related to the release of toxic metals to natural environment might be significantly lower than in case of landfilling. On the other hand, the addition of E-Cat typically decreases rheological properties of fresh cementitious mixes due to its significant water absorptivity and porosity^11,37^. Nevertheless, this effect might be minimized by applying superplasticizer^37^. More detailed review regarding applicability of E-Cat in cementitious materials might be found in the already published Authors’ review paper, see the Ref^43^.

Besides the research on re-using E-Cat wastes as cement or traditional concrete component, some works indicates that those catalyst might be utilized in geopolymer synthetic binders^24,44^. Other minor applications include the use of E-Cat as catalysts for plastic and biomass pyrolysis and gasification or in synthetic fuels manufacturing, as noted in^18^. Moreover, in the future E-Cat wastes will represent an promising source of trace elements, such as lanthanum, of which the recovery process from E-Cat particles was described in^21^. Nevertheless, this paper focuses on cement and concrete as their value chains operate almost everywhere around the world which increase the chance for re-using E-Cat in building materials sector.

### The contribution of research work to the state-of-art literature findings

Despite the encouraging results on the applicability of E-Cat in cement and concrete exhibited on the laboratory scale, there are still some obstacles which deny its use in building materials on industrial scale. This is mainly caused by the variations in the chemical composition, contaminants and performances of E-Cat wastes between various oil refineries where it is generated. Such variations are mainly attributable to the fact that each cracking unit processes specific crude oil fractions on different scale, using individual technology and different process conditions^45^. Hence, the impact of incorporation of specific E-Cat as a cement constituent on the properties of cementitious composites needs to be addressed individually for each type of E-Cat depending on refinery supplying it^45^. Table 1 presents the differences in chemical composition of E-Cat wastes derived from oil refineries from listed countries for the purpose of individual studies conducted by abovementioned research teams cited earlier.

**Table 1:** Oxide composition of E-Cat wastes derived from various worldwide refineries.

Oxide	E-Cat waste from worldwide oil refineries					
[wt%]	Taiwan	Spain	Spain	Oman	China	Lithuania
Ref	^11^	^14^	^37^	^38^	^39,41^	^40^
Na_2_O	0.42	0.29	0.50	0.43	n.d.	0.44
MgO	0.41	0.07	< 0.01	0.26	0.71	0.81
Al_2_O_3_	41.12	49.0	46.00	37.68	38.5	63.43
SiO_2_	51.69	41.7	48.2	39.21	50.1	28.71
P_2_O_5_	n.d.	0.26	n.d.	n.d.	n.d.	n.d.
SO_3_	0.48	n.d.	0.04	n.d.	n.d.	0.55
CaO	1.73	0.19	< 0.01	0.05	n.d.	n.d.
TiO_2_	n.d.	1.00	n.d.	n.d.	n.d.	1.56
Fe_2_O_3_	1.08	1.28	0.95	0.66	1.37	2.28
CuO	n.d.	n.d.	n.d.	n.d.	n.d.	0.95
K_2_O	0.06	0.16	< 0.01	0.06	n.d.	n.d.
La_2_O_3_	n.d.	2.50	n.d.	n.d.	n.d.	n.d.
Cr_2_O_3_	n.d.	0.04	n.d.	n.d.	n.d.	n.d.
SrO	n.d.	< 0.01	n.d.	n.d.	n.d.	n.d.
BaO	n.d.	0.02	n.d.	n.d.	n.d.	n.d.
MnO	n.d.	0.01	n.d.	n.d.	n.d.	n.d.
LOI	3.31	3.70	1.5	2.43	1.2	1.26

Accordingly, it might be seen that catalysts differ regarding the SiO_2_/Al_2_O_3_ ratio, amount or presence of secondary oxides and the value of loss on ignition (LOI), which express the amount of coke deposits. Taking the above into account, an individual approach, supported by the complex research plan is required in terms of re-using waste cracking catalyst from specific oil refinery in construction sector. Moreover, some of the crucial properties of cementitious composites incorporating E-Cat were not thoroughly or at all researched. Among them, the Interphase Transition Zone (ITZ) between E-Cat grains and cement matrix and its porosity caused by E-Cat addition still reveal the field for expansion.

Consideringly, the present paper focuses on the individual studies on the preliminary effect of application of E-Cat originated by Polish oil refinery company as novel SCM, substituting 0–20% of OPC in cementitious blends, on the performances of cement paste mixes prepared out of them. For each OPC substitution level, cement paste mixes were prepared at three different w/c ratios of 0.30, 0.35 and 0.40 and subsequently tested regarding both fresh and hardened state performances. The novelty of the research is underlined by the fact that Polish E-Cat has been not yet investigated in the literature for the following application, especially that, as shown in the subsequent section of the paper, possesses different oxide composition in comparison with catalysts listed earlier in Table 1. Moreover, for the purpose of E-Cat’s pozzolanicity evaluation, besides the already practiced procedure (Chapelle test), the newly developed rapid, relevant and reliable (R^3^) test of pozzolanic reactivity^46^ was utilize in this paper for the first time for this material. Finally, to fill the research gap in terms of ITZ and porosity of cementitious matrices, the hardened samples were analyzed using the SEM JEOL microscope equipped with BSE detector. The porosity associated with microcracks caused by E-Cat addition was evaluated based on SEM images through the dedicated programs ImageJ and Wolfram Mathematica. The cement pastes were decided to be subjected for this measurement as provide the clearest insight into the cementitious matrix due to the lack of any aggregates presence there. The limitation of the current research is the environmental aspect of leaching behavior of heavy metals from cement pastes comprising E-Cat which will be presented by the Authors in the future works.

## Materials and methods

### Materials used

The E-Cat tested during the investigation program was generated by Polish petroleum industry. The OPC type CEM I 52.5 R. provided by Cemex company was used to blend it with E-Cat. This type of cement was chosen as do not contain any addition of other SCMs (such as fly ash, limestone or slag) hence provide better insight for the isolated effect of E-Cat addition on the performances of cementitious composites. The specification of the OPC used in the experiments is in line with the requirements of EN 197-1^28^ in terms of its performances. The chemical composition of those constituents was evaluated applying the X-ray fluorescence (XRF) apparatus Supermini 200 supplied by Rigaku company. The XRF chemical composition is presented in Table 2 with the respective data regarding oxides ranges of E-Cat wastes from worldwide oil refineries presented earlier in the Introduction part.

**Table 2:** XRF-chemical composition of CEM I and E-Cat.

Oxide	Material		
[wt%]	E-Cat tested	E-Cat data range from literature	CEM I 52.5 *R*
Na_2_O	0.37	0.29–0.50	n.d.
MgO	2.2	0.07–0.81	1.74
Al_2_O_3_	43.31	37.68–63.43	6.17
SiO_2_	43.91	28.71–51.69	20.64
P_2_O_5_	0.75	0- 0.26	n.d.
SO_3_	0.12	0.04–0.55	7.93
CaO	0.11	0.05–1.73	62.39
TiO_2_	0.91	0–1.56	n.d.
Fe_2_O_3_	0.59	0.66–2.28	n.d.
K_2_O	n.d.	0.01–0.16	1.12
La_2_O_3_	2.14	0–2.50	n.d.
Cr_2_O_3_	0.02	0.04	n.d.
V_2_O_5_	0.16	n.d.	n.d.
LOI	5.4	1.20–3.31	n.d.

**Fig. 2:**
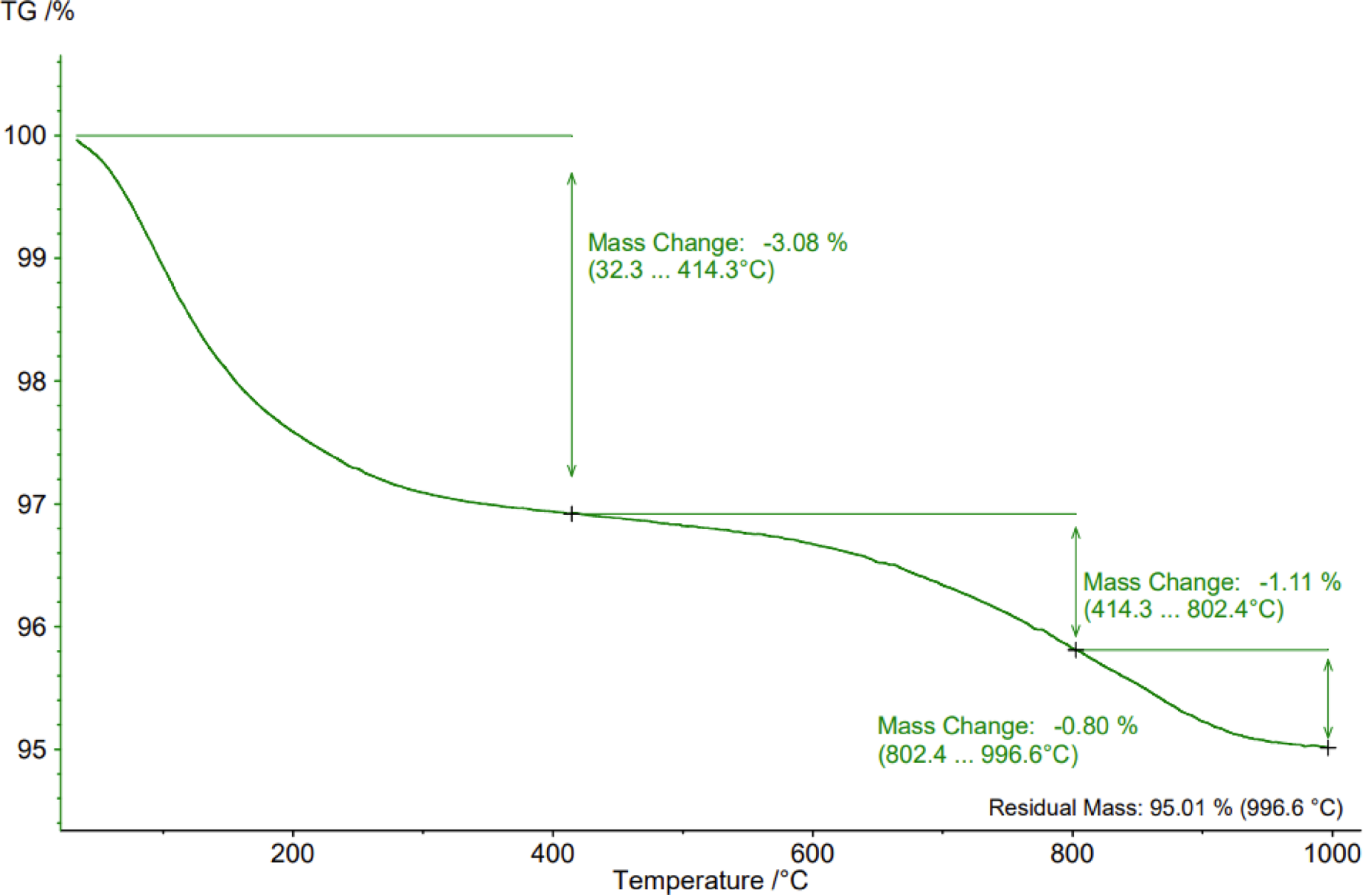
TG plot for E-Cat.

Accordingly, main constituents of E-Cat are reactive Al_2_O_3_ and SiO_2_, representing equally more than 87% of its total mass, supplied by faujasite zeolite and alumina matrix. However, E-Cat tested in this research possesses exceedingly more MgO than catalysts from literature data. Moreover, some oxides such as P_2_O_5_, V_2_O_5_, Cr_2_O_3_ and La_2_O_3_, were detected in case of tested E-Cat, otherwise to literature data, excepting^14^. Finally, Polish E-Cat is characterized by the highest LOI value of 5.40, which corresponds to the unburnt coke deposits on its surface and inside pores. Such variables are in line with findings presented in the Ref^45^. and push the need of individual approach for researching E-Cat wastes. The 5.40% LOI value was correlated well with the result of thermogravimetry (TG) analysis of Polish E-Cat carried out until 1000 °C in Argon atmosphere. The TG plot is presented in Fig. 2.

**Fig. 3:**
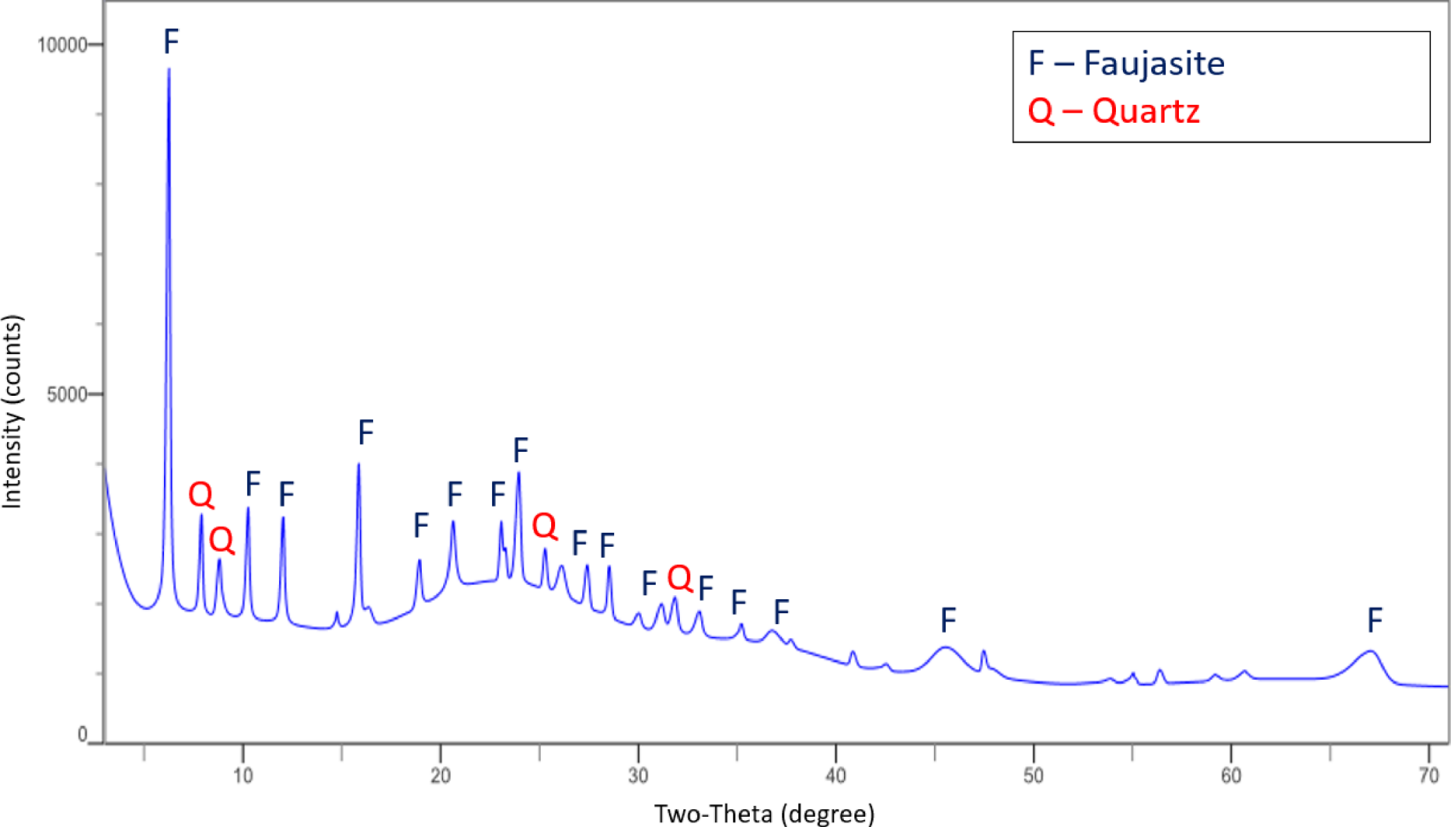
XRD diffractogram of E-Cat.

Accordingly, the three main mass changes were detected associated with the decomposition of hydrocarbons. According to the paper^47^ presenting the decomposition temperature ranges of hydrocarbons typically converted in FCC process, the second peak might be associated with decomposition of gas oil fractions, paraffines and aromatic structures while the last change might be caused by decomposition of long-chain heavy gas oil fractions or aromatics, characterized by more stable structures hence enhanced decomposition temperature. The first peak might be attributable to boiling of lighter hydrocarbons, even the residual of cracking products remaining on the surface of E-Cat, such as hydrocarbons with up to 10–15 carbon atoms in the chain and to the evaporation of inter-package water from the faujasite structure (detected on XRD). The mineralogical composition of E-Cat was measured on X-ray diffraction (XRD) device Miniflex 600 produced by Rigaku company equipped with a lamp with copper anode operating in the wavelength of 3–72°. The respective diffractogram is presented in Fig. 3. Both the TGA and XRD analysis were done on dried E-Cat grains ground on ring mill and sieved on 63 μm sieve without any additional treatment.

As it was expected, among the minerals detected in E-Cat powder were mainly crystalline zeolite (Faujasite - F) and silicate parts of the E-Cat (Quartz - Q). The characteristic elevated background of diffractogram is attributable to the amorphous content of the zeolite phases and presence of organic matters which correlates with the TG curve and loss on ignition of E-Cat. However, the crystal structures from XRD do not cause any signal in TG apart from potential evaporation of inter-package water from the faujasite structure (decomposition up to 400 °C). The XRD results are in line with the analysis presented in the article^48^.

**Fig. 4:**
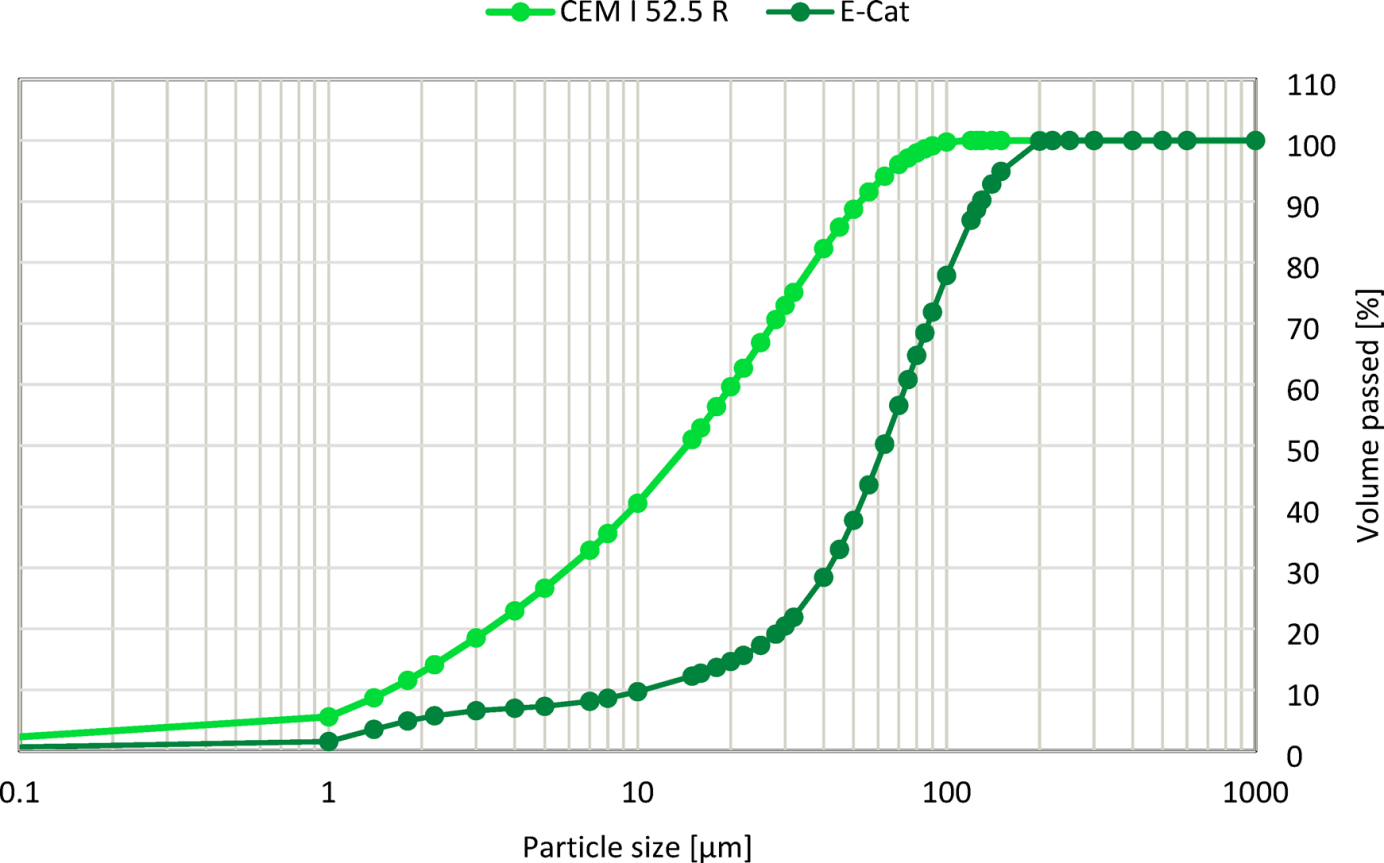
PSD pattern of cement and E-Cat.

Figure 4. Illustrates the Particle Size Distribution (PSD) pattern of cement and E-Cat. PSD measurement was done using the MasterSizer Scirocco 2000 device.

**Fig. 5:**
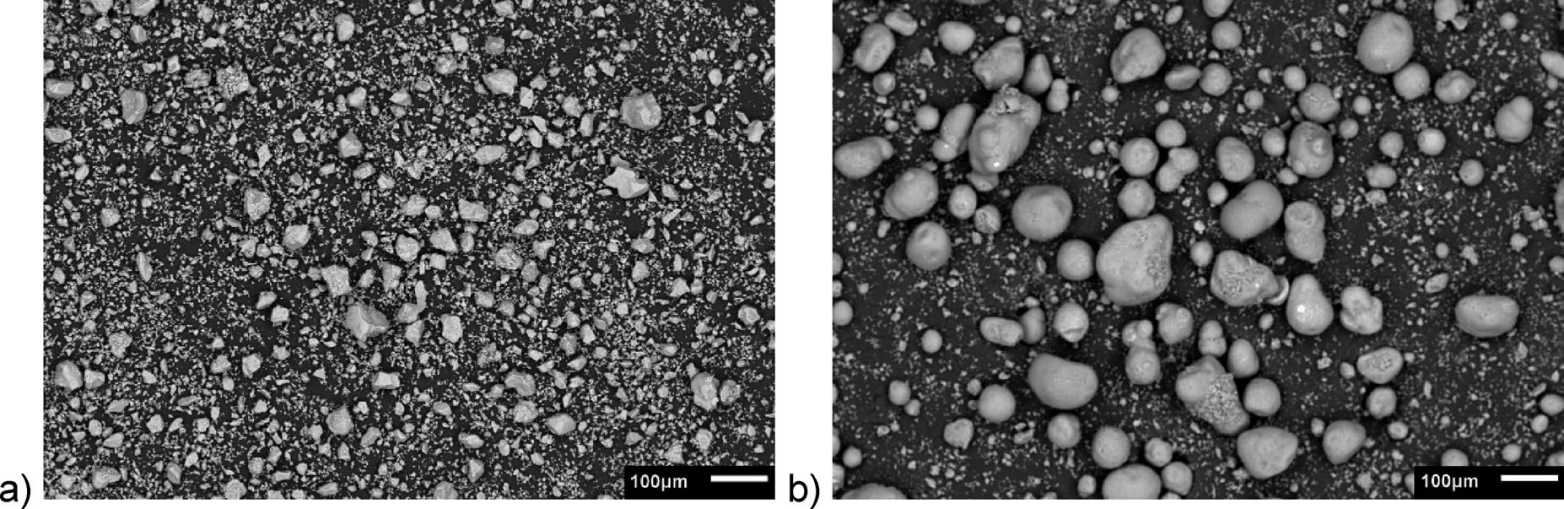
SEM images of the grains of materials used in research: (**a**) cement; (**b**) E-Cat.

Consideringly, the E-Cat particles are characterized by d(0.1), d(0.5) and d(0.9) equal to 10.56, 62.73 and 129.24 μm, respectively, while the ones of cement by d(0.1), d(0.5) and d(0.9) equal to 1.58, 14.46 and 52.52 μm, respectively. Therefore, E-Cat grains were found to be generally coarser than cement ones. Such finding was confirmed by the Scanning Electron Microscope (SEM) images of grains of tested CEM I 52.5 R and E-Cat taken at ×100 magnification, presented in Fig. 5.

### Mixes formulations and samples Preparation

The raw ingredients, i.e. OPC and E-Cat, were mixed and homogenized for 20 min in the laboratory turbula mixer to prepare the homogenous blends with different percentages of OPC substituted with spent catalyst. ranging from 0 to 20%, by the mass of OPC. Once it was done, each blend was mixed in the laboratory bowl with distilled water at three different w/c ratios of 0.30. 0.35 and 0.40, according to the EN 196-3^49^ standard for cement paste mixing (two 90 s. mixing periods separated by 30 s. break for scraping of the paste from the bowl and stirrer). Note that total mass of blend (sum of OPC and E-Cat) was the same for all mixes.

The resulted cement paste mixes were described as X% E-Cat, where X referred to amount of E-Cat present in the blend, and assigned to the w/c ratio used to mix the paste. The reference mix, consisting of plain OPC was named as Ref for each w/c ratio. Table 3 shows the formulations of every cement paste. calculated for 1 dm^3^ of the mix.

**Table 3:** Cement paste mixes prepared during research.

w/c ratio	Material	Paste name				
		Ref	5% E-Cat	10% E-Cat	15% E-Cat	20% E-Cat
0.30	OPC, kg	1.593	1.508	1.423	1.340	1.257
	E-Cat, kg	0.000	0.079	0.158	0.236	0.314
	Distilled water, kg	0.478	0.476	0.474	0.473	0.471
0.35	OPC, kg	1.475	1.397	1.319	1.242	1.165
	E-Cat, kg	0.000	0.073	0.146	0.219	0.291
	Distilled water, kg	0.516	0.515	0.513	0.511	0.510
0.40	OPC, kg	1.374	1.301	1.229	1.157	1.086
	E-Cat, kg	0.000	0.068	0.137	0.204	0.272
	Distilled water, kg	0.550	0.548	0.546	0.545	0.543

After the consistency tests. the mixes were casted into the molds with dimensions of 40 × 40 × 160 mm^3^ and 40 × 40 × 40 mm^3^ for the preparations of samples for further analysis. After 24 h samples were demolded and placed in tap water until the measurement day.

### Testing methods

#### Consistency tests

**Fig. 6:**
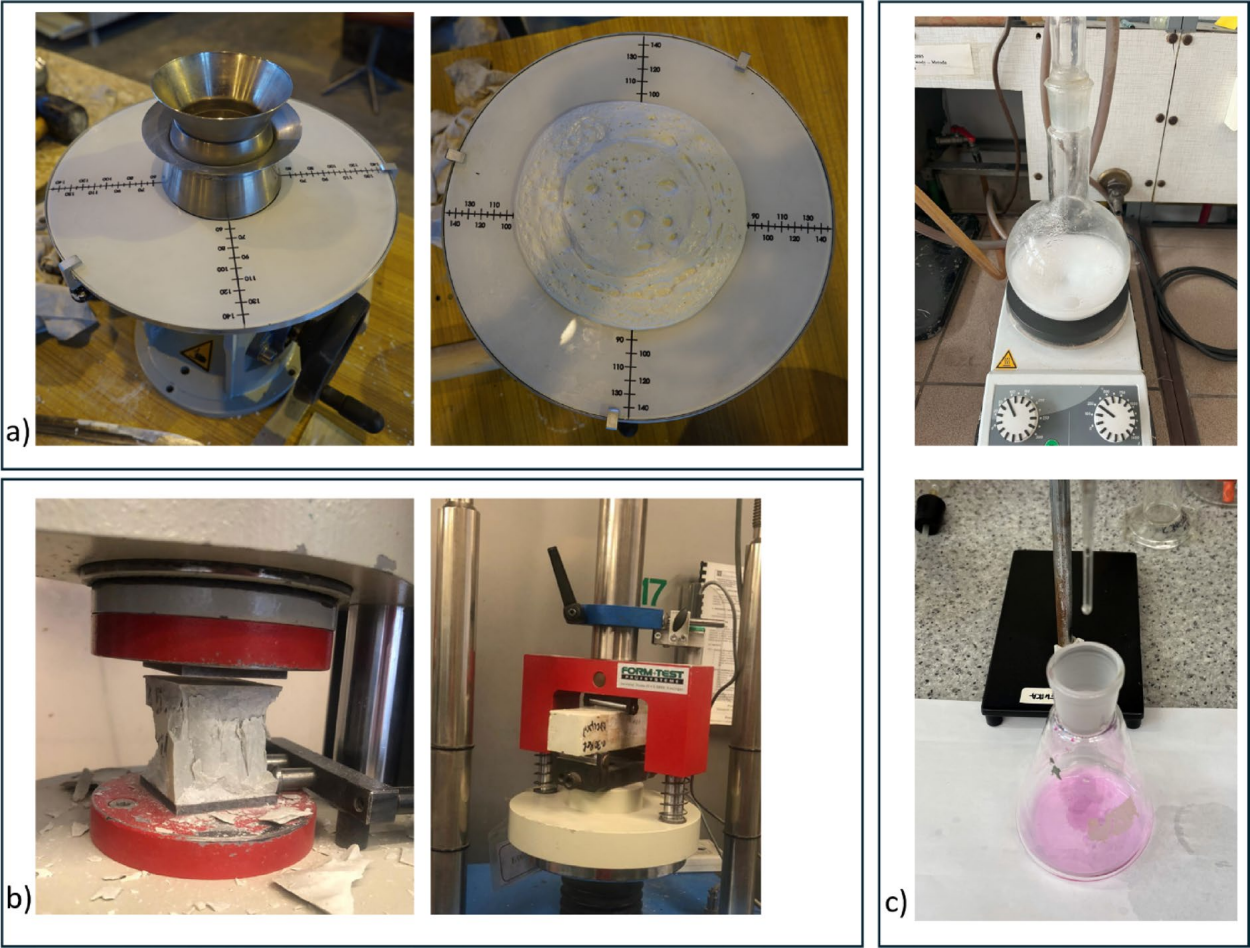
Visualization of testing methods: (**a**) consistency test; (**b**) mechanical tests; (**c**) pozzolanic activity Chapelle test.

Consistency of mixes was investigated by flow table test, according to EN 1015-3^50^. The cement paste mix was filled into the mold on the spreader disc and tamped with tamper. The excessed mix was removed. The mold was lifted and the disk was dropped for 15 times inside 15 s. The two perpendicularly diameters of the spread mix were determined and their arithmetic average was calculated as spread of the mix (see Fig. 6a)).

#### Flexural strength tests

The flexural strength was tested on beam samples. Three samples prepared from each mix were crushed after 7 and 28 curing days using automatic compression machine (see Fig. 6b)), according to EN 196-1^51^ standard.

#### Compressive strength tests

The compressive strength was tested on halves of beam samples after the flexural strength measurement. As such, the number of six samples from each mix were crushed after 7 and 28 curing days under loading rate of 1.5 MPa/s using automatic compression machine (see Fig. 6b)), according to EN 196-1^51^ standard.

#### Water absorptivity tests

The water absorptivity of hardened paste samples was measured after 28 hardening days as a difference between masses of samples before and after putting in the vessel filled with water for 24 h. Before the measurement, samples were dried to constant mass, while after removing from the vessel, the uncombine water was eliminated from their surfaces by a cloth. The water absorptivity was calculated by dividing this mass difference by the mass of dried sample before putting in water and presented in percentages.

#### Microstructure analysis

The microstructure of cement paste samples was evaluated using the Scanning Electron Microscope (SEM) JEOL model JSM-6610 A and material contrast mode of BSE detector operating in material-contrast mode. The observations were carried out at an accelerating voltage of 20 kV, a beam current of 39 nA and a working distance ranging between 10 and 25 mm, depending on the depth of the analyzed region. Sample preparation involved cutting the specimens to expose a fresh cross-section, followed by grinding with abrasive papers of 320–1200 grit. Subsequently, the surfaces were polished using diamond suspensions of 6 μm and 1 μm. To prevent surface charging during imaging and to ensure adequate electrical conductivity, a narrow copper strip was applied along the edge of each specimen, and the samples were coated with a 30–40 nm carbon layer deposited by thermal evaporation under high vacuum (2 × 10⁻⁵ Pa).

The SEM images obtained from the polished sections were used to assess both microcracks and pores within the cementitious matrix. Porosity quantification was based on the procedure described by Wong et al.^52^, adapted here to the type of images analyzed. First, each micrograph was imported into ImageJ and converted into a greyscale histogram. From this distribution, a cumulative greyscale histogram was generated. The threshold level required for image binarization was identified from the first inflection of the cumulative curve. Following the approach of Wong et al.^52^, the inflection point was determined by constructing two tangents: one fitted to the initial, nearly linear portion of the curve, and the other to the first noticeable curvature. The intersection of these two lines represents the location of the inflection point, which was taken as the binary threshold.

All pixels with greyscale values between 0 (black) and the threshold level were classified as voids, corresponding to pores or microcracks. ImageJ was then used to generate binary images, where voids appear as black pixels. Final porosity values were calculated in Wolfram Mathematica by counting the number of black pixels and dividing this value by the total number of pixels in the image.

To construct the plots presented in Fig. 15, the binary images were further analyzed column by column. In each vertical pixel column, the proportion of black pixels relative to the total pixel count was calculated, allowing the distribution of porosity across the full width of the specimen to be visualized.

#### Pozzolanic reactivity evaluation

##### Chapelle test

The pozzolanic reactivity was investigated through the Modified Chapelle Test for pozzolanic materials, standard by NF P 18–513^53^. Accordingly, two lime solutions, consisting of 2 g of lime saturated in 250 ml of water, were prepared in parallel in laboratory flasks. Then, 1 g of waste catalyst was placed in one flask and both flasks were placed in the same heating plates with magnetic stirrers at 90 °C, continuously stirring, for 16 h. Afterwards, solutions were cooled to ambient conditions and stirred for 30 additional minutes after the saccharose solution was added. Then, about 200 ml of each solution was filtered and exactly 25 ml of resulted filtrates were titrated with 0.1 M HCl under phenolphthalein. The result of measurement was assumed as the mass of Ca(OH)_2_ in mg reacted with 1 g of E-Cat, calculated from the stoichiometry of titration reaction through the difference in amount of HCl sufficient to titrate sample with and without E-Cat (see Fig. 6c)).

##### Rapid. Relevant and reliable (R^3^) test

The R^3^ developed procedure was also utilize for the pozzolanic reactivity evaluation of the E-Cat. The procedure was designed for various pozzolans, such as calcined clays and is described in the standard EN 196 − 12 method A^54^. The synthetic paste mixes consisting of SCM (E-Cat), Ca(OH)_2_ and limestone in the mass ratio of 1:3:0.50 were prepared, homogenized and dissolved in water containing potassium basis (water to solid ratio of 1.2). The reactivity was evaluated based on the heat release of the synthetic paste using the eight-channel heat flow isothermal calorimetry TAM Air for 7 days at 40 °C. The description of the procedure is well explained in^46^.

The visualization of certain testing methods applied in this research is presented in Fig. 6, while the summary of associated standards is tabled in Table 4.

**Table 4:** Summary of associated standards applied in the experimental program of the research.

Material		
Experimental phase	Testing method/measurement	Associated standard
Raw materials investigation	Chemical composition (XRF)	EN 196-2 ^55^
Raw materials investigation	Thermogravimetry analysis (TGA)	Internal procedure
Raw materials investigation	Mineralogical composition (XRD)	EN 13925-2 ^56^
Raw materials investigation Raw materials investigation Raw materials investigation	Particle Size Distribution (PSD) Chapelle test Rapid. relevant and reliable test	Internal procedure NF P 18–513 ^53^ EN 196 − 12 method A ^54^
Mix preparation and testing	Mix preparation	EN 196-3 ^49^
Mix preparation and testing	Consistency test	EN 1015-3 ^50^
Hardened paste testing	Samples preparation and curing	EN 196-1 ^51^
Hardened paste testing	Flexural/compressive strength	EN 196-1 ^51^
Hardened paste testing	Water absorptivity	Internal procedure
Hardened paste testing	Microstructure and porosity	Internal procedure

## Results

### Consistency tests

**Fig. 7:**
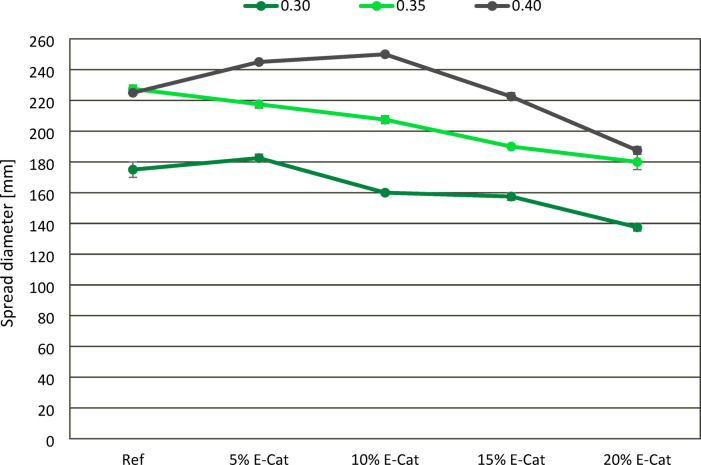
Effect of E-Cat on consistency of cement paste mixes.

The effect of E-Cat on consistency of cement paste mixes was presented in Fig. 7. As it was expected, the consistency of mixes was significantly affected by w/c ratio. The more water was used to prepare mixes, the higher spread diameters were measured between mixes with the same amount of E-Cat. In general, the increase of incorporation of E-Cat tended to constantly decrease the fluidity of subsequent cement paste mixes for w/c ratios of 0.30 and 0.35, apart from the mix containing 5% E-Cat at w/c ratio of 0.30. For those series, mixes with the highest amount of E-Cat, i.e. substituting 20% of OPC, revealed comparable decrease of spread diameter, i.e. for 21.4% and 20.9%, in comparison with the adequate Ref values, i.e. 175.0 mm and 227.5 mm, for w/c ratios of 0.30 and 0.35, respectively. When 0.40 w/c ratio was applied, the consistency values of mixes were comparable or even slightly more fluid than the Ref value (225.0 mm), when E-Cat substituted up to 15% of OPC. However, when E-Cat was substituting 20% of OPC, the resulted mix revealed decrease of spread diameter for 16.7% in relation to Ref value. Overall. it might be concluded that the addition of E-Cat tends to reduce the consistency. This effect is strengthen by two factors: low w/c ratio and high and OPC substitution level higher than 10–15% by mass. Such impact correlates with the specific surface area of E-Cat grains which provides its vital absorptivity. Nevertheless, it might be reduced or outweighed by tailoring mix formulation and application of compactible superplasticizer^37^.

### Compressive strength tests

**Fig. 8:**
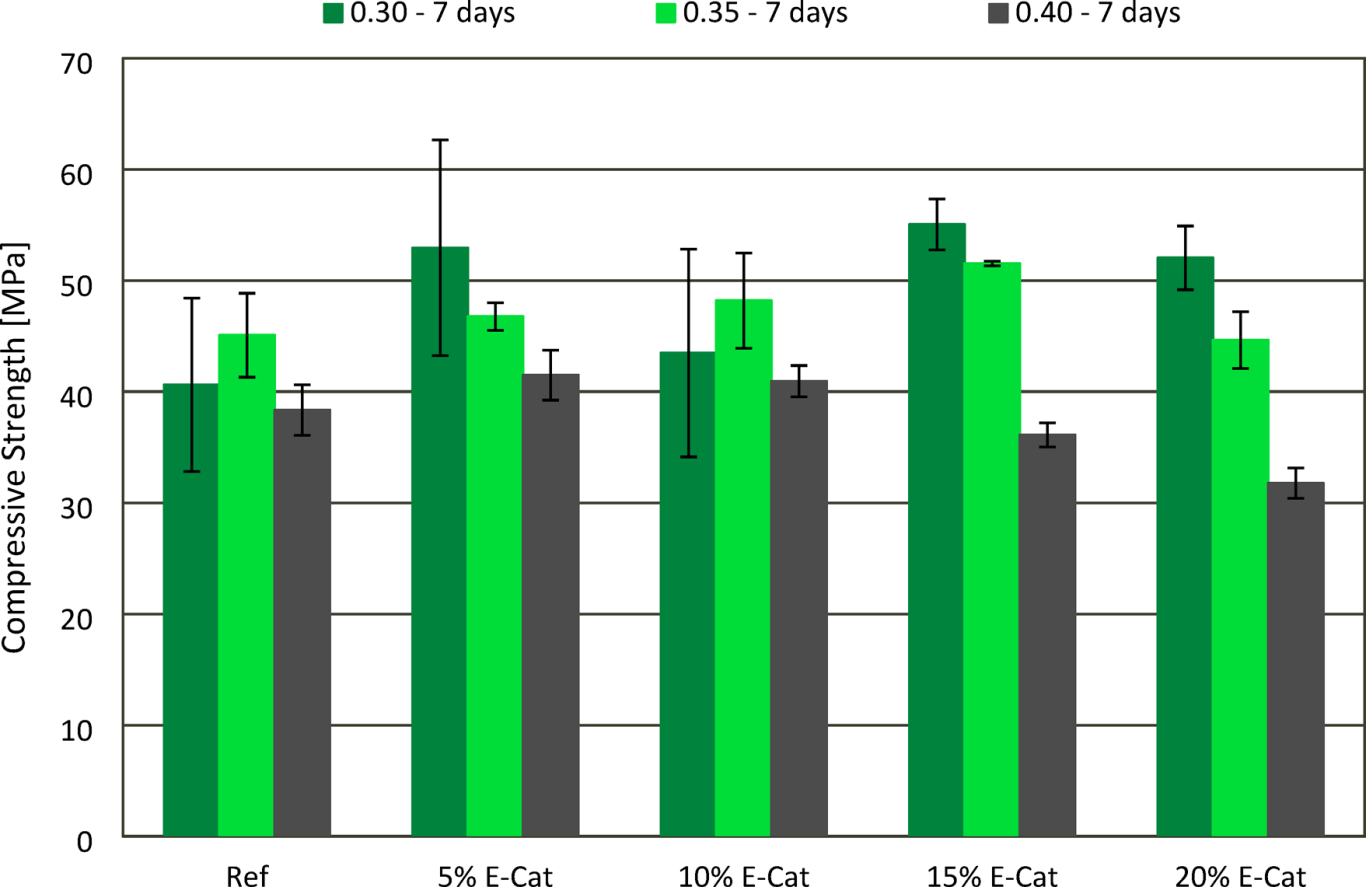
Effect of E-Cat on compressive strength of paste samples after 7 days of curing.

**Fig. 9:**
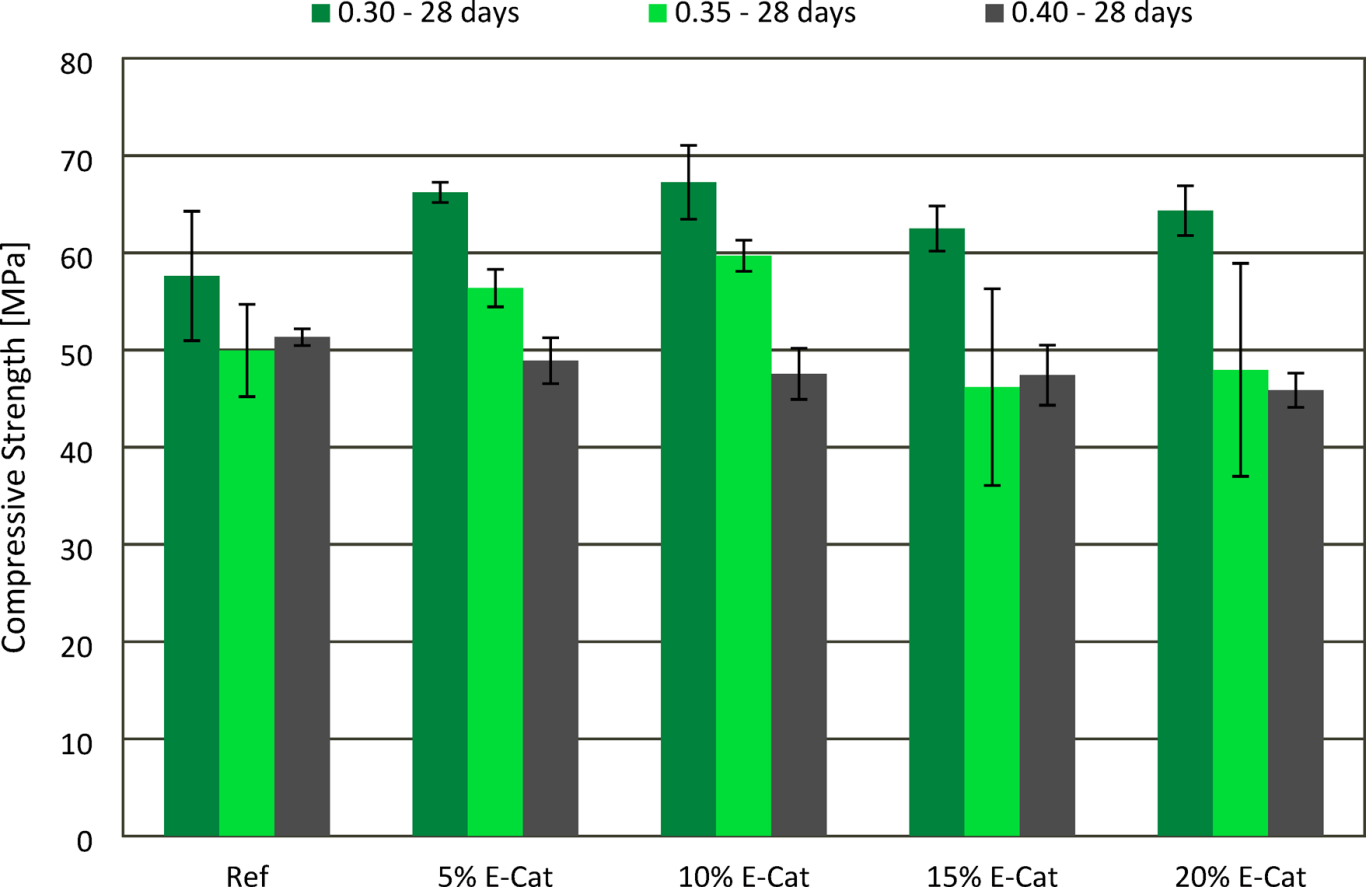
Effect of E-Cat on compressive strength of paste samples after 28 days of curing.

The effect of E-Cat on CS of hardened paste samples after 7 and 28 curing days was presented in Figs. 8 and 9, respectively. The final testing period of 28 days, despite brings about some limitation in terms of long-term E-Cat effect on mechanical properties, was chosen as it is stated in the requirements for cement standard (EN 197-1^28^ as the standard testing period for compressive strength measurement. In most cases, both 7 and 28 days compressive strength (CS) values increased with decreasing w/c ratio between pastes with the same amount of E-Cat incorporated. When w/c ratio of 0.30 was used, CS values of each sample with incorporation of E-Cat were higher than Ref values both after 7 (40.6 MPa) and 28 (57.6 MPa) days. The increasement of 7 days CS was vary from 7.1 to 35.7%, measured for 10% E-Cat and 15% E-Cat, respectively, while the one of 28 days CS, from 8.5 to 16.7%, measured for 15% E-Cat and 10% E-Cat sample, respectively, when compared to respective Ref values. The similar tendency of results were found for w/c ratio of 0.35, mentioning that CS of samples containing E-Cat exceeded Ref, however only until no more than 15 and 10% of OPC was substituted, in case of measurements after 7 and 28 days, respectively. The highest CS values were obtained for 15% E-Cat and 10% E-Cat, respectively exceeding Ref value for 14.2 and 19.6% after 7 and 28 days. Further incorporation of E-Cat tended to slightly decrease CS values in comparison with Ref, after 7 (45.1 MPa) and 28 (49.9 MPa) days of curing, respectively. For 0.40 w/c, only 5% E-Cat and 10% E-Cat samples revealed enhancement of 7 days CS in relation to Ref (38.3 MPa), exciding its value for 8.4 and 6.8%, respectively. Nonetheless, 28 days CS values were found to decrease constantly in accordance with increasing OPC substitution level. This regression was varied from 4.7 to 10.5%, measured for 5% E-Cat and 20% E-Cat samples, respectively, in relation to Ref value (51.3 MPa). It is vital that even if some of the samples prepared with w/c ratios of 0.35 and 0.40 revealed deterioration of either 7 or 28 days CS, in each case these decreases were lower than respective substitution levels of OPC would indicate for.

### Flexural strength tests

**Fig. 10:**
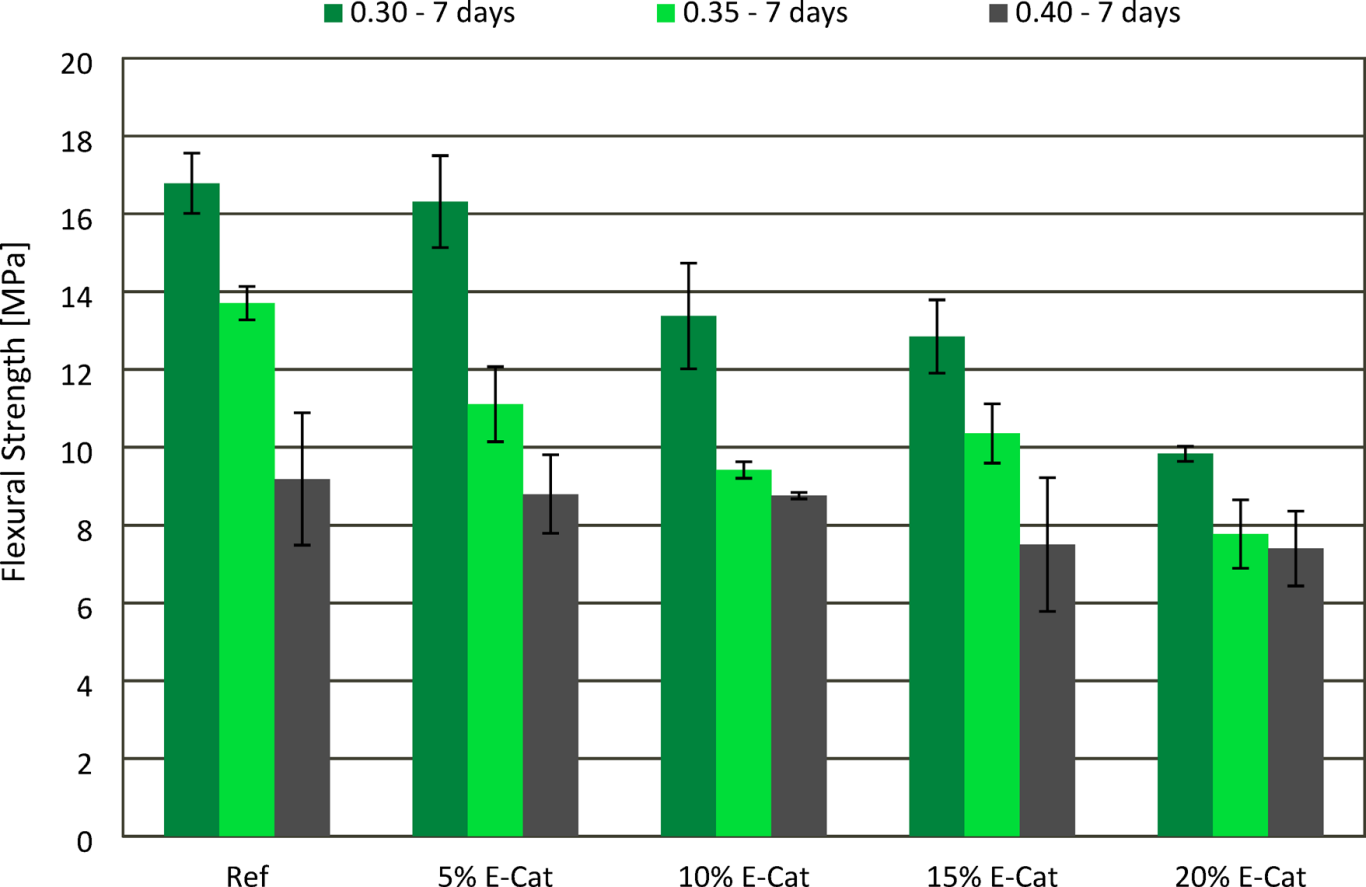
Effect of E-Cat on flexural strength of paste samples after 7 days of curing.

**Fig. 11:**
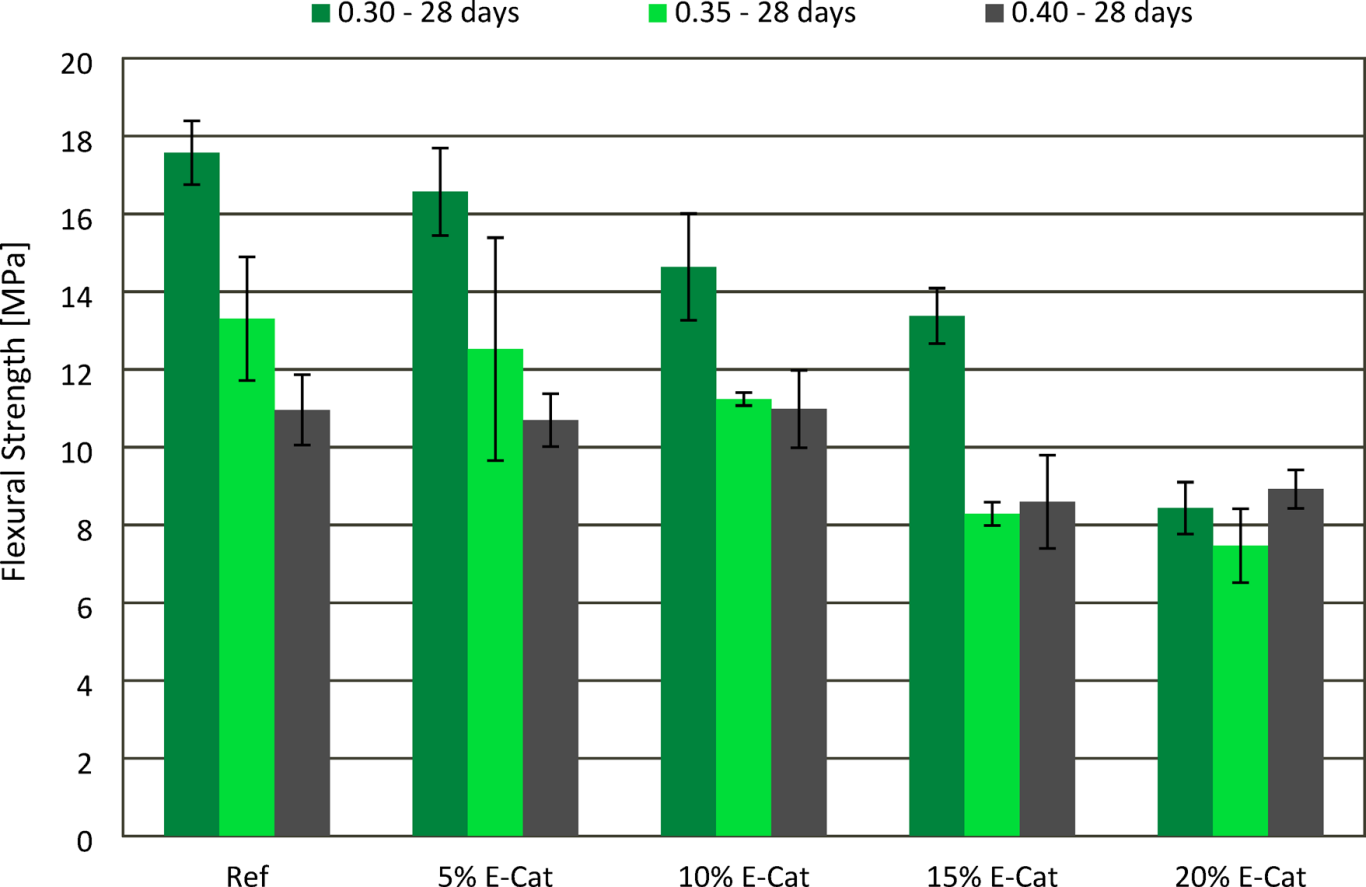
Effect of E-Cat on flexural strength of paste samples after 28 days of curing.

The effect of E-Cat on FS of hardened paste samples after 7 and 28 curing days was presented in Figs. 10 and 11, respectively. Similar to the evaluation of CS, in most cases, both 7 and 28 days flexural strength (FS) values revealed increasement with decreasing w/c ratios between pastes with the same amount of E-Cat incorporated. When 0.30 w/c ratio was used, samples showed constant deterioration of both 7 and 28 days FS in accordance with increasing amount E-Cat incorporated. This accounted for 7 days FS regression from 3.0 to 41.7%, as well as 28 days FS from 5.7 to 52.3%, 5% E-Cat and 20% E-Cat samples, respectively, in relation to 7 (16.8 MPa) and 28 (17.6 MPa) days FS of Ref samples. In case of 0.35 w/c ratio, the similar tendency was observed as all samples with E-Cat addition showed FS deterioration in relation to respective values of Ref samples, both after 7 (13.7 MPa) and 28 (13.3 MPa) days. However, 7 days FS of 15% E-Cat sample was found to be out of this decreasing tendency, presenting higher FS than 10% E-Cat sample. The 28 days FS values tended to decrease constantly in accordance with the increasing OPC substitution level, in this case, from 6.0 to 43.6% for 5% E-Cat and 20% E-Cat samples, respectively, in relation to Ref value. For samples with the highest w/c ratio of 0.40, both 7 and 28 days FS values were noticed to be the same or comparable as adequate Ref values, when no more than 10% of E-Cat was incorporated. For both curing ages, more significant decrease of FS was observed for 15% E-Cat samples, i.e. for 18.4 and 21.8%, while compared to Ref values after 7 (9.2 MPa) and 28 (11.0 MPa) curing days, respectively. Further substitution of OPC, i.e. until 20%, did not cause further significant effect on FS.

### Water absorptivity tests

**Fig. 12:**
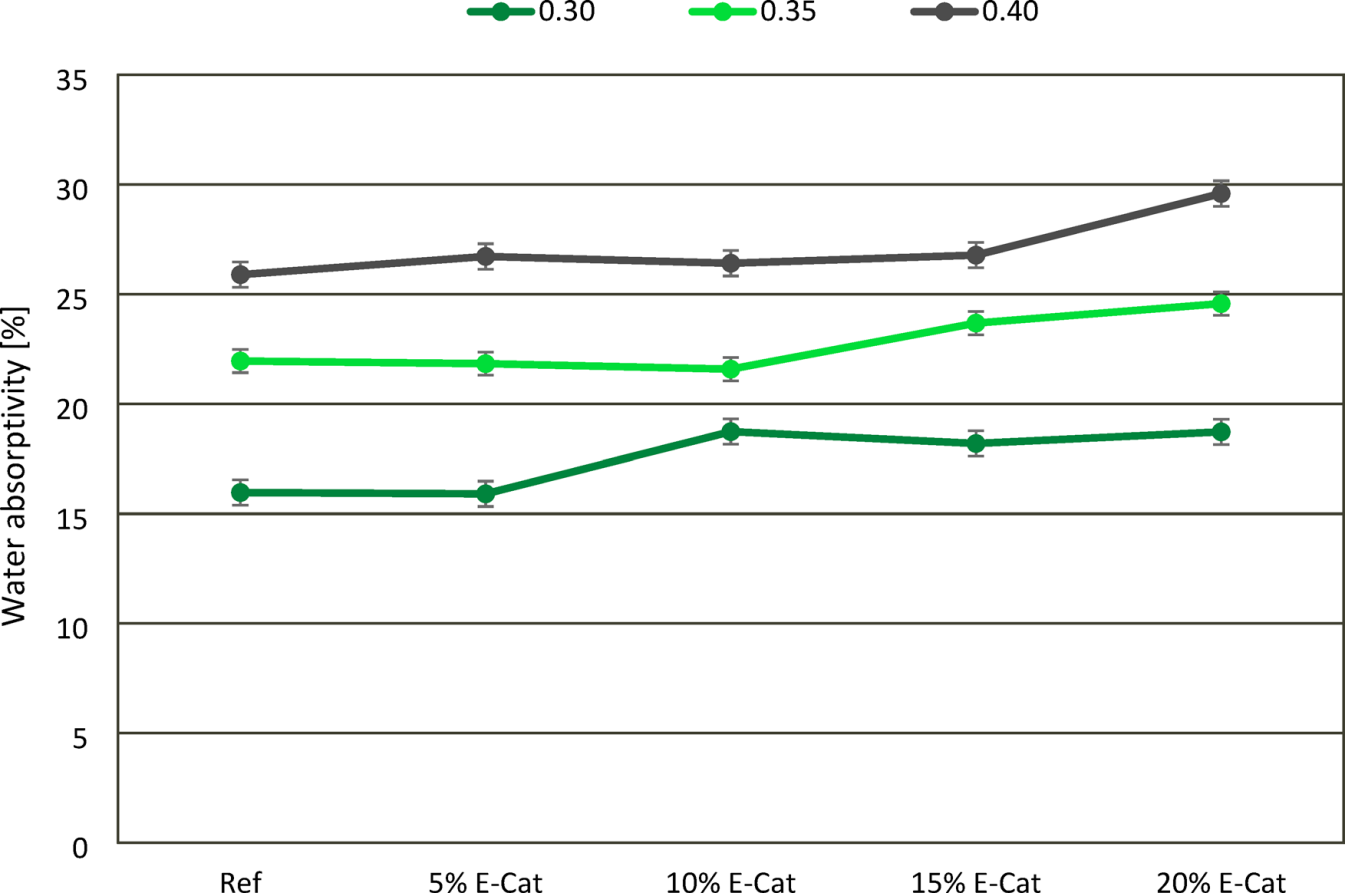
Effect of waste FCC catalyst on water absorptivity of paste samples.

The effect of E-Cat on water absorptivity of hardened paste samples was presented in Fig. 12. In contrast to the tendencies investigated for CS and FS measurements, water absorptivity values were found to increase with the increasement of w/c ratio between series with same amount of E-Cat incorporated. It is caused by the reduction of capillary pores and microcracks resulted from the evaporated excessive water from the beams with the reducing w/c ratio. It was proved that samples prepared with w/c ratios of 0.30, 0.35 and 0.40 exhibited comparable water absorptivity to the respective reference samples, when no more than 5, 10 and 15% of OPC was substituted by E-Cat, respectively. Thereby, more significant increase in water absorptivity was investigated for 10% E-Cat, 15% E-Cat and 20% E-Cat samples, respectively at 0.30, 0.35 and 0.40 w/c ratio, exceeding adequate Ref values for 17.3, 7.9 and 14.3%. Overall, it was clearly indicated that the addition of E-Cat generally tends to increase water absorption, while tailoring w/c ratio might delay or mitigate this effect with respect to cement substitution ratio. This phenomena is directly associated with the fact that E-Cat, due to its highly porous nature, contributes to increasement of porosity and microcracks in the cementitious matrices, especially in the Interphase Transition Zone (ITZ) located in its unreacted grains (if appear). This conclusion was outlined in the next part of this paper.

### Microstructure analysis

**Fig. 13:**
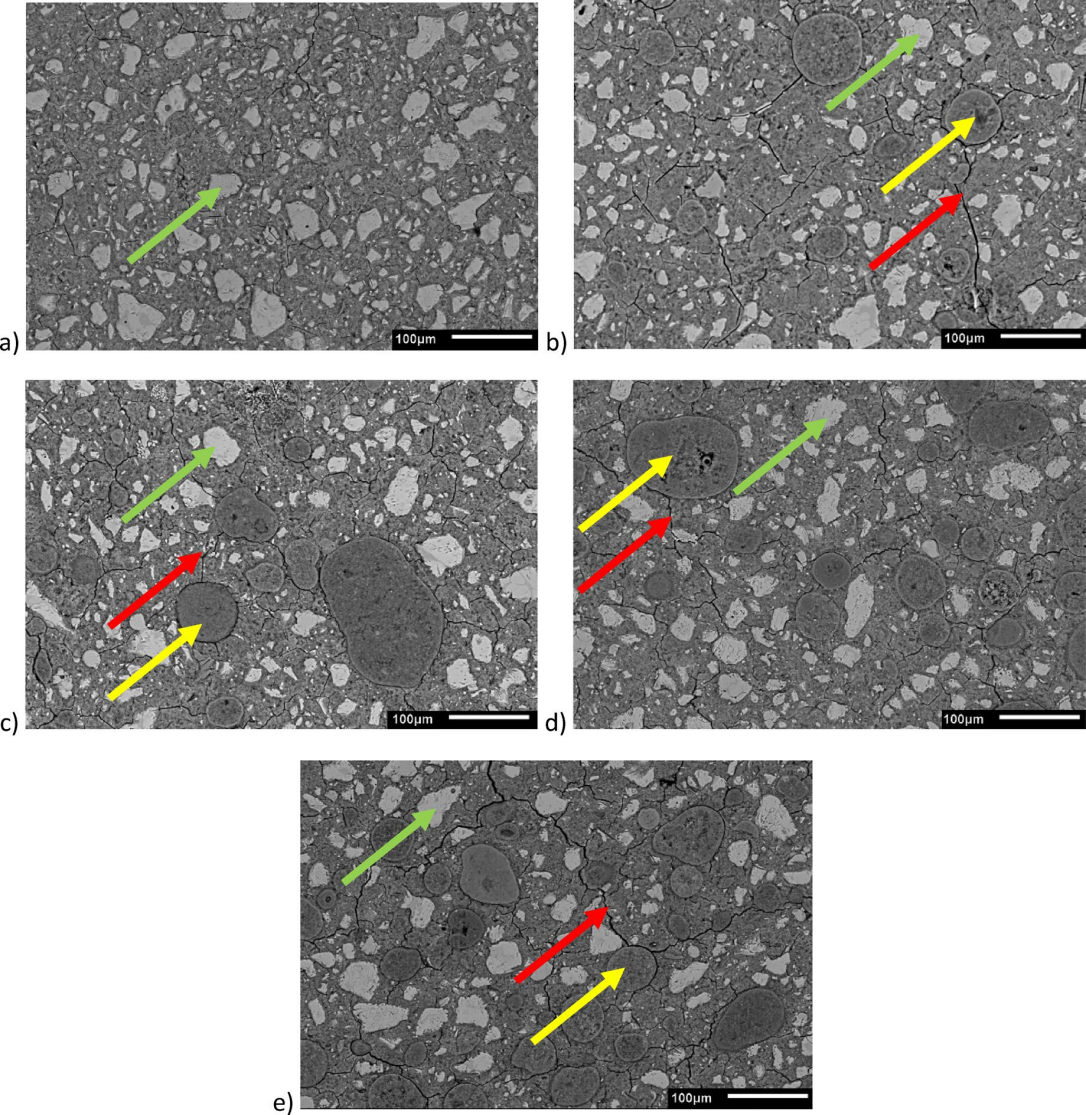
Microstructural images of cementitious matrices at 0.30 w/c obtained by SEM microscope: (**a**) Ref; (**b**) 5% E-Cat; (**c**) 10% E-Cat; (**d**) 15% E-Cat; (**e**) 20% E-Cat.

The microstructures of cementitious matrices of samples prepared with w/c ratio of 0.30 were presented in Fig. 13, as SEM images taken at magnification ×200. The microstructure of Ref sample is presented in Fig. 13a), while the subsequent letters of b), c), d) and e) correspond to samples with 5, 10, 15 and 20% of OPC substituted with E-Cat, respectively. The main aim of these analyses was to demonstrate effect of E-Cat on porosity of cementitious matrix. Therefore, images were taken for pastes prepared with w/c ratio of 0.30 as they contain the lowest amount of capillary water in the system and hence their capillary porosity is limited to minimum, providing better insight into microcracks resulting from the presence of the spent catalyst. The light grey shapes are attributed to unreacted cement grains (see green arrows). The spherical shapes in darker color and coarser dimensions are referred to unreacted E-Cat particles (see yellow arrows). The microcracks appear as dark, elongated features (see red arrows), while all the surrounding is attributed to hydrated cement phases comprising secondary C-S-H phase from pozzolanic impact of E-Cat (in case of samples containing E-Cat). Accordingly, the microstructure of Ref sample was found to be well-compacted and homogenous. Hence, all the resulted microcracks in subsequent samples with the incorporation of E-Cat might be mainly determined by its presence in the system. It was observed that most microcracks originated in the Interfacial Transition Zone (ITZ) between unreacted particles of catalyst and cementitious hydrates surrounding them, as presented below.

**Fig. 14:**
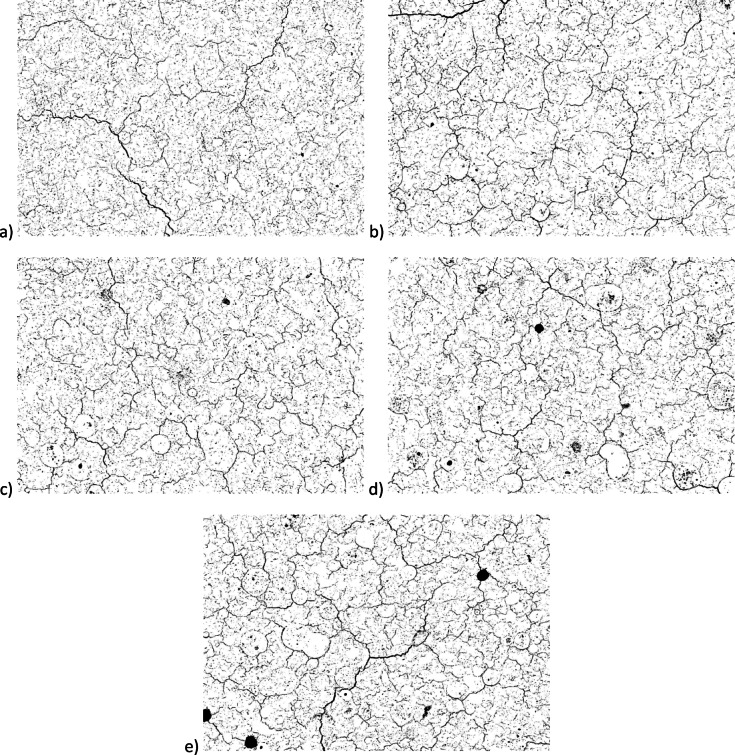
Binary images showing the void structure of related cementitious matrices: (**a**) Ref; (**b**) 5% E-Cat; (**c**) 10% E-Cat; (**d**) 15% E-Cat; (**e**) 20% E-Cat.

To provide better sight for these microcracks, ImageJ program was used to create pores images, presenting them as black pixels in Fig. 14. It should be noted that the binarization procedure identifies all voids present in the matrix, including both pores and microcracks, therefore the generated images represent the total void structure rather than microcracks alone. As previously, Fig. 14a) refers to Ref sample, while the subsequent letters of b), c), d) and e) correspond to samples with 5, 10, 15 and 20% of OPC substituted by E-Cat.

**Fig. 15:**
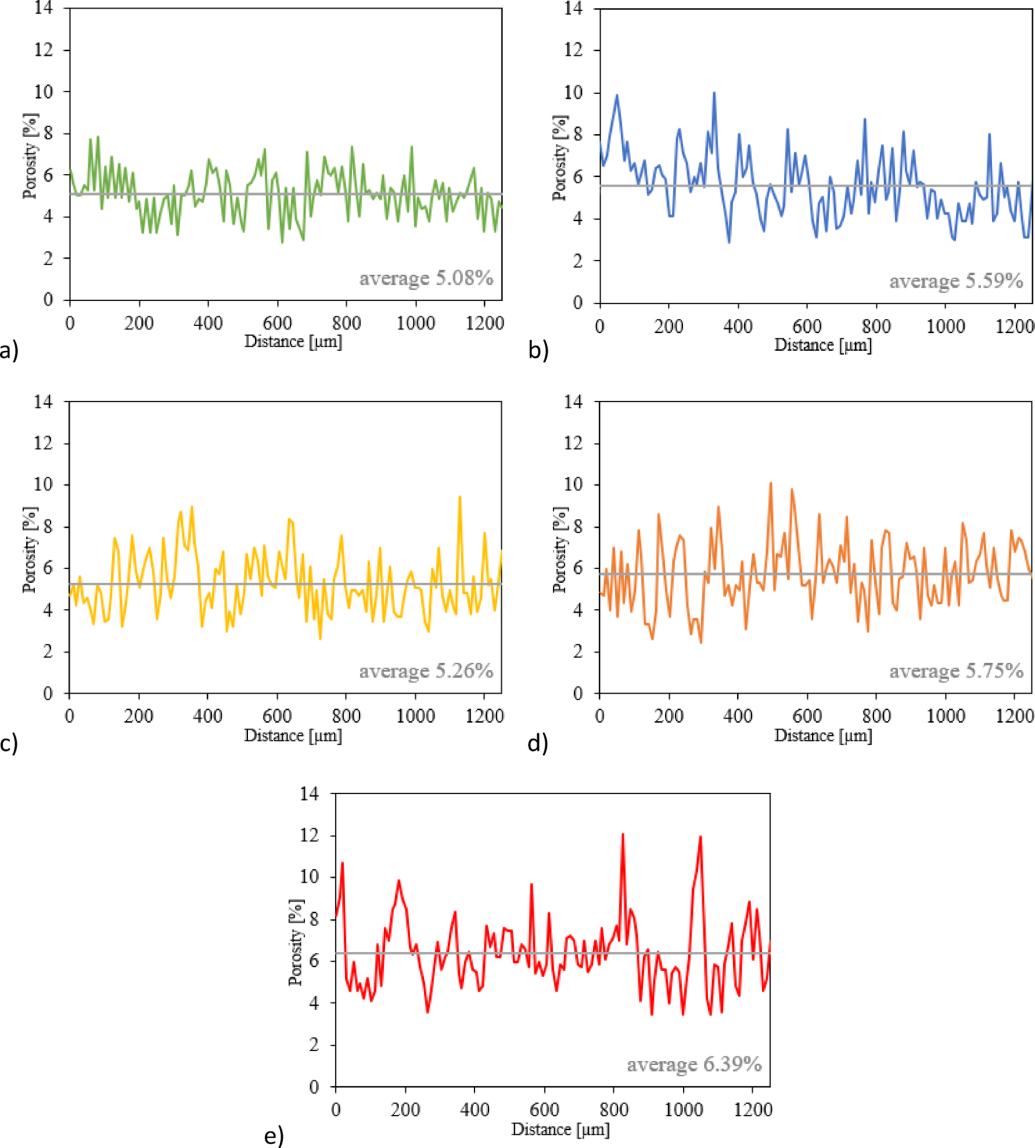
Percentage pore content for the related cementitious matrices: (**a**) Ref; (**b**) 5% E-Cat; (**c**) 10% E-Cat; (**d**) 15% E-Cat; (**e**) 20% E-Cat.

Subsequently, the Wolfram Mathematica program was employed to calculate average pores percentages for each cementitious matrix based on the above microcracks images. The pore percentage was calculated by dividing the number of black pixels by the total number of pixels in the image, following the procedure described by Wong et al.^52^. The resulting plots with the percentage porosity values were presented in Fig. 15. The percentage porosity values were evaluated as 5.08%, 5.59%, 5.26%, 5.57% and 6.39% for the samples: Ref, 5% E-Cat, 10% E-Cat, 15% E-Cat and 20% E-Cat, respectively, described by the subsequent letters of a), b), c), d) and e). The slight decrease observed at 10% E-Cat represents a small local deviation, while the overall evolution from 5.08% (Ref) to 6.39% (20% E-Cat) clearly demonstrates the increasing void content with higher catalyst replacement. The lowest porosity value was obtained for the Ref sample, which exhibited a homogeneous and well-compacted matrix.

### Pozzolanic activity

The direct pozzolanic activity of E-Cat was evaluated using two different tests, as described in the methodology section, i.e. Chapelle test and rapid, relevant and reliable (R^3^) test. The principal of those tests is to mimic the pozzolanic reaction of SCM with Portlandite which occurs in real cement system using the synthetic blends of E-Cat and Ca(OH)_2_ (and limestone in case of R^3^). The power of this reaction, i.e. pozzolanic activity, is measured by the consumption of Ca(OH)_2_ expressed in mg/g of E-Cat and by the heat release inside 7 days expressed in J/g of E-Cat, respectively for Chapelle and R^3^ test. According to Chapelle test, 1 g of E-Cat was found to consumed 870.3 mg of Ca(OH)_2_, while according to R3 test, 1 g of E-Cat released the heat of 497 J.

## Discussion

Analyzing the experimental results, it was found that the amount of E-Cat used to prepare cementitious mixes affected their fresh and hardened state performances tested during this research for each w/c ratio. In general, the similar tendency of impact of E-Cat addition on each tested properties was observed for all w/c ratios.

The increasing incorporation of E-Cat generally tended to reduce the consistency of mixes, especially for higher OPC substitution levels. This deterioration is caused by the fact that E-Cat is a highly porous material with significant water demand. Hence, it reduces the free-water available in cementitious system as and hence rheological performances of mixes. These findings are in accordance with the other investigation carried out on E-Cat originated in various European or worldwide oil refineries, presented in many papers, such as^11,14,37^. However, this negative effect might be compensated by applying superplasticizer to the cement mix as pointed out in both in literature studies^37^ and by the previous Author’s experiments^57^. The slight increase of spread diameter observed for certain mixes with 5 or 10% of E-Cat in the blend was assumed to be resulted from measurement statistic as neither technical nor scientific explanations have been found there, especially that the nano-filling effect improving consistency was denied as particles of E-Cat are coarser than cement.

The incorporation of up to 20% of E-Cat enhanced both early-stage and long-term compressive strength of hardened cement pastes. The most vital enhancement of 7 and 28 days compressive strength compared to reference samples was 35.5% and 16.7%, respectively, investigated for the samples containing 15% and 10% E-Cat, respectively, both at 0.30 w/c ratio. Such phenomenon is caused by pozzolanic activity of E-Cat, i.e. its reaction with Portlandite (Ca(OH)_2_) which resulted in secondary C-S-H gel formed in cementitious matrix and was found to be in accordance with other results from the literature, presented in e.g^11,37,38^.

Pozzolanicity of Polish E-Cat was confirmed directly, as each 1 g of E-Cat consumed 870.3 mg of Ca(OH)_2_ and released the heat of 497 J in Chapelle and R^3^ test, respectively. For comparison, other pozzolans, such as metakaolin and fly ashes consume between 560 and 1140 and 540–725 mg of Ca(OH)_2_/g, respectively, according to the paper^42^. Despite this, in the same work^42^ E-Cat was found to consumed almost doubled amount of Ca(OH)_2_, i.e. 1540 mg. Nevertheless, the R3 test result for E-Cat, despite not being measured in the literature previously in case of this material, shows its significant pozzolanicity, as for comparison to E-Cat heat release of 497 J/g, according to the literature, natural or calcined pozzolans released from 50 to 300 J/g and fly ashes from 160 to 360 J/g^58^.

Despite this, deterioration of compressive strength values in relation to reference samples was observed for certain samples prepared at 0.35 or 0.40 w/c, especially those, which were prepared of composite cements with higher OPC substitution levels. The possible reason for that is the dilution effect, which predominates the pozzolanic reaction, especially when the limit of cement content leads to insufficient amount of Portlandite formed in the hydration from the stoichiometry viewpoint of pozzolanic reaction in the whole system or in case of local available Portlandite content. The following conclusion was also stated in the paper^41^. Nonetheless, none of the tested sample revealed more significant CS deterioration that respective cement reduction level would indicate for, which indirectly shows that pozzolanic impact of E-Cat predominates over the dilution effect in the system.

Taking into consideration the microstructure it might be concluded that incorporation of E-Cat resulted in the formation of additional pores, identified mainly in the Interphase Transition Zone (ITZ) between their unreacted particles and cementitious hydrates surrounding them, indicating poor adhesive properties between those phases. Therefore, these cementitious matrices were found to be less homogeneous and more cracked than the one of reference cement paste. This effect was strengthened especially for samples containing higher amount E-Cat as more unreacted catalyst’s particles might be also resulted from non-availability of sufficient amount of Portlandite available to react with them, especially locally, if E-Cat grains are not spread regularly in the paste volume. Moreover, as E-Cat has high water demand^11^, it might act as a storage for some portion of water initially, releasing it in the later stage and hence produce microcracks which contributed to enhanced porosity. Thus, samples with higher amount of E-Cat revealed higher percentage porosity values. Overall, the content of microcracks could be reduced by the further reduction of w/c ratio which might be achieved successfully by the application of compatible superplasticizing admixture, as presented in^37^ or, in case of construction site, specialized curing conditions, such as application of water on the surface of concrete.

The more porous microstructure of cementitious matrices was correlated with the loss of flexural strength of hardened samples. This deterioration was also more significant when higher amounts of OPC were substituted with E-Cat. Therefore, negative effect of E-Cat addition on flexural strength of pastes was associated with these pores formed.

Finally, the resulted microcracks also enhanced the water absorptivity of hardened samples, providing more spaces for water absorption. Higher water absorptivity of samples might be also associated with the significant water absorption of E-Cat and its porosity^11^ as its unreacted particles might provide additional water demand, which was noticed in the work^59^.

Overall, according to the results of preliminary investigation which evaluated the possibilities of re-using E-Cat derived from Polish petroleum industry as pozzolanic SCM, this material was concluded to be a promising additive for cementitious blends, substituting up to 10–20% of OPC, depending on w/c ratio. The summarized tendency of impact provided by incorporation of E-Cat on the performances of cementitious pastes tested in this research compared to reference pastes was illustrated in Table 5. 

Finally, the cumulative impact was calculated individually for each mix formulation as the sum of percentage change of each parameter. Note that the opposite value of percentage change of water absorptivity was used for calculation, as the enhancement of this parameter brings about negative impact to concrete and vice versa, especially from the durability perspective.

**Table 5:** Summarized tendency of impact provided by incorporation of E-Cat on the investigated properties of cement pastes tested in this research.

w/c	Investigated property	Mix description and percentage change of parameters			
		5% E-Cat	10% E-Cat	15% E-Cat	20% E-Cat
**0.3**	Spread diameter	4.3	-8.6	-10	-21.4
	7 days compressive strength	30.3	7.0	35.5	28.1
	28 days compressive strength	14.9	16.7	8.5	11.7
	7 days flexural strength	-2.8	-20.3	-23.5	-41.4
	28 days flexural strength	-5.7	-16.7	-23.9	-52
	Water absorptivity	-0.4	17.3	13.9	17.2
	**Cumulative impact**	**41.4**	**-39.2**	**-27.3**	**-92.2**
**0.35**	Spread diameter	-4.4	-8.8	-16.5	-20.9
	7 days compressive strength	3.7	6.9	14.3	-1.0
	28 days compressive strength	12.9	19.5	-7.5	-4.0
	7 days flexural strength	-19	-31.3	-24.4	-43.3
	28 days flexural strength	-5.9	-15.5	-37.7	-43.9
	Water absorptivity	-0.5	-1.7	7.9	11.9
	**Cumulative impact**	**-12.2**	**-27.5**	**-79.7**	**-125.0**
**0.4**	Spread diameter	8.9	11.1	-1.1	-16.7
	7 days compressive strength	8.2	6.8	-5.8	-17.1
	28 days compressive strength	-4.7	-7.4	-7.6	-10.7
	7 days flexural strength	-4.2	-4.6	-18.3	-19.4
	28 days flexural strength	-2.4	0.2	-21.5	-18.6
	Water absorptivity	3.2	2.0	3.4	14.3
	**Cumulative impact**	**2.6**	**4.1**	**-57.7**	**-96.8**

According to the table above, the formulation 0.3 5% E-Cat revealed the most positive effect in terms of cumulative impact on the tested performances of cement paste with respect to the reference plain cement paste. The positive cumulative impact was also noted for the following formulations 5% E-Cat and 10% E-Cat at w/c 0.4. The rest of formulations revealed negative cumulative impact, mainly due to the significant reduction of consistency (spread diameter) and flexural strength with increasing E-Cat content. Nevertheless, those performances might be overweighed by the tailoring the formulation using the superplasticizer and, as example, fibers. Overall, the promising results obtained in this work underline the correctness of the proposed research direction. i.e. to investigate the possibility of re-using E-Cat from Polish petrochemical industry as a green resource for cement and concrete production. The results were confirmed by the available literature regarding their tendency and correlated with the performances of E-Cat. However, this research work was conducted in case of primary cement composites, i.e. pastes. As such, ITZ between the improved cementitious gel containing more C-S-H phases and aggregate grains, which might be crucial in terms of potential impact on mechanical, physicochemical, and microstructural performances of cementitious composites, could not been investigated. Therefore, the Authors plan to extend the scope of this work in the future in case of more advanced cementitious composites comprising aggregate, such as cement mortars. Besides promising results, the additional motivation to carry out the research is the fact that E-Cat has not been thoroughly researched in the literature regarding the applicability in construction sector yet. As such, some research gaps are still not filled, especially in case of the correlation between the performances of E-Cat in purified and impurified form the impact it provides to cement material, particularly in terms of its potential toxicity and immobilization of heavy metals. Moreover, some of the other oil refinery wastes have been not investigated according to the literature. To fill these gaps, apart from E-Cat, some the other waste materials from oil refinery, such as spent Claus catalyst and ceramic balls from molecular sieve residues are planned to be researched. All the materials will be investigated both in the impurified and purified, adapting chemical and thermal method, forms, to investigate if these processes might additionally valorize waste materials from oil refinery and consequently improve their performances in terms of the applicability in cement production, regarding the properties and toxicity of cement mortars prepared with their addition. Such research direction emphasizes the innovative approach to the worldwide-scale problem of oil refinery wastes disposal and, as already mentioned, give grounds to bring about high contribution to the development of science in the field of recycling of waste materials in cement industry.

## Conclusions

This paper focused on the preliminary investigation of the effect of applicability of E-Cat from Polish petrochemical industry as novel SCM material, substituting 0–20% of OPC, by mass, in cement paste mixes on their fresh and hardened state performances at three w/c ratios of 0.30, 0.35 and 0.40. The main conclusions were pointed out from the presented research work:

1. E-Cat generated by Polish petroleum industry differs with E-Cat wastes from worldwide refineries in terms of oxide composition. Polish catalyst possesses more MgO (2.2% in contrast to maximum 0.81% from the literature review) and La_2_O_3_ (detected only in case of Spanish E-Cat apart from the current research). Moreover, some other minor oxides were detected for Polish E-Cat in contrast to the worldwide E-Cats from the literature studies (e.g. V_2_O_5_). Polish E-Cat is characterized by higher loss on ignition value (5.4% in context to maximum 3.31% in literature). Due to the presence of more than 87% of SiO_2_ and Al_2_O_3_, Polish E-Cat successfully undergoes the reaction with Portlandite which confirms its suitability to be a promising SCM for cement substitution in cementitious materials. Despite the Chapelle test result for Polish E-Cat was found to be lower than maximum result in literature (870.3 mg of Ca(OH)_2_/g in contrast to 1540 mg of Ca(OH)_2_/g for Portuguese E-Cat), still it overcame the 630 mg of Ca(OH)_2_/g (which is an adopted criteria in the standard to consider a material as pozzolanically active). Nevertheless, the R^3^ result (497 J/g of E-Cat) was found to be an excellent result, exceeding the results for other SCMs drastically.
2. According to research results, the incorporation of E-Cat as a substitute of up to 5–20% of OPC, depending of w/c ratio, resulted in the enhancement of 28 days compressive strength (crucial properties of cement composites) of hardened cement pastes for up to 16.7%, without scarifying deterioration of their other performances. The comparable results were found in the literature. However, addition of Polish E-Cat brings about some microcracks which contributed to the enhanced porosity of the paste microstructures. When 5% of E-Cat was used at 0.30 w/c ratio, the most significant positive effect in terms of cumulative impact on the tested performances of cement paste was observed.
3. The pozzolanic reactivity of E-Cat was confirmed by different tests, i.e. Chapelle test and R^3^. Both results indicated its vital pozzolanicity, which confirm the suitability of the use of R^3^ method for such type of waste materials, which was not previously applied in the literature.

## Data Availability

Data will be made available on request.
